# Identification of Traditional Medicinal Plant Extracts with Novel Anti-Influenza Activity

**DOI:** 10.1371/journal.pone.0079293

**Published:** 2013-11-27

**Authors:** Dhivya Rajasekaran, Enzo A. Palombo, Tiong Chia Yeo, Diana Lim Siok Ley, Chu Lee Tu, Francois Malherbe, Lara Grollo

**Affiliations:** 1 Environment and Biotechnology Centre, Faculty of Life and Social sciences, Swinburne University of Technology, Hawthorn VIC, Australia; 2 Sarawak Biodiversity Centre, Kuching, Sarawak, Malaysia; Centers for Disease Control and Prevention, United States of America

## Abstract

The emergence of drug resistant variants of the influenza virus has led to a need to identify novel and effective antiviral agents. As an alternative to synthetic drugs, the consolidation of empirical knowledge with ethnopharmacological evidence of medicinal plants offers a novel platform for the development of antiviral drugs. The aim of this study was to identify plant extracts with proven activity against the influenza virus. Extracts of fifty medicinal plants, originating from the tropical rainforests of Borneo used as herbal medicines by traditional healers to treat flu-like symptoms, were tested against the H1N1 and H3N1 subtypes of the virus. In the initial phase, in vitro micro-inhibition assays along with cytotoxicity screening were performed on MDCK cells. Most plant extracts were found to be minimally cytotoxic, indicating that the compounds linked to an ethnomedical framework were relatively innocuous, and eleven crude extracts exhibited viral inhibition against both the strains. All extracts inhibited the enzymatic activity of viral neuraminidase and four extracts were also shown to act through the hemagglutination inhibition (HI) pathway. Moreover, the samples that acted through both HI and neuraminidase inhibition (NI) evidenced more than 90% reduction in virus adsorption and penetration, thereby indicating potent action in the early stages of viral replication. Concurrent studies involving Receptor Destroying Enzyme treatments of HI extracts indicated the presence of sialic acid-like component(s) that could be responsible for hemagglutination inhibition. The manifestation of both modes of viral inhibition in a single extract suggests that there may be a synergistic effect implicating more than one active component. Overall, our results provide substantive support for the use of Borneo traditional plants as promising sources of novel anti-influenza drug candidates. Furthermore, the pathways involving inhibition of hemagglutination could be a solution to the global occurrence of viral strains resistant to neuraminidase drugs.

## Introduction

Influenza viruses are highly infective and constitute a major causative agent for recurrent epidemics and pandemics. On average, about 10% of the world's population is infected by the virus annually, resulting in around 250,000 deaths, hence posing a serious health threat [1]. More generally, the viruses cause acute respiratory infections referred to as “flu” and hospitalizations represent a considerable financial burden upon the global economy.

Influenza viruses are classified under the family Orthomyxoviridae and are divided into three types: A, B and C. The genomes of type A and B consist of eight segments of negative-sense single-stranded RNA and the virions express two major surface glycoproteins, haemagglutinin (HA) and neuraminidase (NA). Conversely, Type C contains seven RNA segments and express only one major surface glycoprotein, hemagglutinin-esterase-fusion (HEF) protein [2]. Amongst the types, A and B are the predominant causes of human infections [3], with Type A being further divided into subtypes, based on the antigenicity of the HA and the NA. To date, 17 HA (H1–H17) and 9 NA (N1–N9) subtypes have been identified, and most subtypes are present in waterfowl and shorebirds [1], [4], [5]. Of these, only H1N1, H2N2 and H3N2 have been associated with pandemics and epidemics in human populations [1]. Types A and B viruses spread globally in pandemics mediated through mutations that generate antigenic drift and shift [6].

Vaccines form the basis for the prevention of influenza infections, yet there are substantial drawbacks. The current preventive strategy involves annual vaccination, requiring regular monitoring to confirm matching between vaccines and the circulating virus strains. Vaccination failures have been widely documented and in the elderly, where most of the mortality occurs, vaccines are only around 50% effective [7].

In the eventuality of a pandemic infection with a new strain, antiviral drugs represent the first line of defence [8]. Currently available anti-influenza drugs aim to block viral replication and spread, thereby resulting in early recovery from the symptoms of flu. First generation influenza antivirals, referred to as ion channel blockers (Amantadine and Rimantadine), act on the viral M2 protein, which is essential for the organized release of nucleocapsid after fusion of the virus with the endosomal membrane [9]. Side effects associated with the central nervous system and the gastrointestinal tract, and the rapid emergence of antiviral resistance during therapy, have limited the usefulness of adamantanes in the prevention and treatment of influenza [10], [11].

As a result, a second generation of anti-influenza drugs, the neuraminidase inhibitors (NAI), were developed. There are currently two NAI drugs approved for use worldwide, Oseltamivir and Zanamivir, and two others approved in North Asia but still in trials elsewhere (Laninamivir and Peramivir) [11]. Zanamivir (GG167), a sialic acid analogue, and Oseltamivir, an ethyl ester derivative of Oseltamivir GS4071, inhibit the sialidase activity of the viral neuraminidase by competitive and irreversible binding to the NA active site [12], [13]. However, there are side effects associated with the administration of Oseltamivir and Zanamivir, such as nausea, vomiting, neuropsychiatric events, abdominal pain, diarrhoea, sinusitis, headache and dizziness. Furthermore, Oseltamivir-resistant H1N1 viruses spontaneously arose and spread globally in 2008 [10]. These data highlight the requirement for a third generation of anti-influenza drugs that would exhibit a different mode of action [8]. Thirteen years after the launch of Zanamivir and Oseltamivir, the quest for unique lead structures remains an area of intensive research [14].

Plants represent one of the important sources of lead compounds, with up to 40% of modern drugs being derived from plant materials. Empirical knowledge based on the ethnomedical benefits of plants, coupled with bioassay-guided fractionation and isolation, has the potential to identify novel antivirals that could be used against influenza. Currently, herb and plant resources are relatively unlimited with respect to the search for functional phytochemicals but these resources are dwindling rapidly due to deforestation and advancements of industrialization. Even though a number of studies have been performed using purified plant chemicals, very few studies have addressed the antiviral activities of crude plant extracts [14], [15].

The search for plant-based antivirals against the influenza virus is promising, as several plants have been shown to possess anti-influenza activity, some of which include: *Thuja orientalis*, *Aster spathulifolius*, *Pinus thunbergia* [16], *Allium fistulosum* [17], *Sambucus nigra* [18] and *Psidium guajava* [19]. Active components have also been isolated from crude plant extracts employing chemical fractionation techniques. Patchouli alcohol isolated from the leaves of *Pogostemon cablin* [20], cardiotonic glycoside obtained from *Adenium obesum* (Forssk.) [21] and polyphenols from the roots of *Glycyrrhiza uralensis* [14] have been shown to exhibit anti-influenza activity. Progress in the field of anti-influenza herbal medicines has provided alternative therapeutic measures for the treatment of influenza virus infection. For instance, two Japanese herbal medicines, Shahakusan and hochuekkito, have been shown to possess *in vivo* activity against influenza virus [22], [23]. On the other hand, studies on Jinchai, a capsule made of Traditional Chinese Medicine, indicated inhibitory activity against viral adsorption and cell membrane fusion, thereby blocking transcription and replication of the virus [24]. Also, Lianhuaqingwen capsule, a natural herbal medicine, was shown to have similar therapeutic effectiveness to that of Oseltamivir, in terms of reducing the duration of illness and viral shedding of Influenza A virus [25].

In this work, we have used a range of bioassays to screen fifty medicinal plant extracts for antiviral activity against influenza Type A viruses. The results demonstrate the anti-influenza potential of some extracts which act via a unique mode of action when compared to the currently available antiviral drugs. In the eventuality of an influenza pandemic, third generation of anti-influenza compounds would be extremely beneficial and the source of such compounds could be medicinal plants.

## Materials and Methods

### Cells

Madin Darby Canine Kidney (MDCK) cells, obtained from the American Type Culture Collection (Manassas, VA) were grown at 37°C with 5% CO_2_ in Roswell Park Memorial Institute medium (RPMI; Invitrogen, No: 22400-105), supplemented with 10% foetal bovine serum (FBS; Invitrogen, No: 16140-071) and 1% Penicillin-Streptomycin (Invitrogen, No: 15140-122). Before adding the compounds or the virus, or when quantifying the results, the monolayers were thoroughly washed twice with phosphate buffered-saline (PBS, pH 7.4 at room temperature). In all experiments, the following controls were included: cell control (cells that were not infected with the virus or treated with the plant extracts), virus control (cells that were infected only with the virus but not treated with the plant extracts in the antiviral assays), and the positive controls (virus-infected cells treated with Zanamivir or Oseltamivir).

### Viruses

Type A influenza virus strains, “Mem-Bel” reassortant (H3N1), a reassortant of A/Memphis/1/71 (H3N2) × A/Bellamy/42 (H1N1), containing the HA of A/Memphis/1/71 and the remaining gene segments of A/Bellamy/42 and A/Puerto Rico/8/34 (H1N1) “PR8” were provided by Professor Lorena Brown, Department of Microbiology and Immunology, The University of Melbourne, Australia. Virus stocks were grown in MDCK cells using RPMI medium supplemented with 4 µg/mL trypsin (Sigma, No: T1426) at 37°C in 5% CO_2_ for three days as described elsewhere [26]_ENREF_16. Supernatants containing virus were collected after cytopathic effects (CPE) were noted and antiviral titres were determined using 50% Tissue Culture Infectious Dose, according to Reed and Muench's endpoint method [27], and a colorimetric endpoint to obtain quantitative results [28]. All aliquots of virus stocks were stored at −80°C until use.

### Plant extracts

Fifty medicinal plant extracts, collected from the tropical rainforests of Borneo, Sarawak, Malaysia, were selected on the basis of their traditional use in healing various diseases, including symptoms of influenza such as cough and sore throat. They were originally extracted with a mixture of dichloromethane and methanol in a 1∶1 (v/v) ratio and subsequently concentrated using a rotary evaporator. The yield is dependent on the part of plant used; around 0.05 to 0.10 g of extract was obtained from 6 g of whole plant or leaves, whereas the yield was reduced to 0.01 to 0.05 g when extracts were obtained from stems and roots. Prior to use, the extracts were reconstituted in PBS with 10% dimethyl sulfoxide (DMSO, SIGMA No: D 5879) and filtered using a 0.45 µm filter (Sartorius Stedium Australia No: 16533K).

### Cytotoxicity studies of extracts

Briefly, MDCK cells were seeded into 96-well flat-bottomed microtitre plates (Costar) at 4×10^3^ cells per well. Following overnight incubation, the media of MDCK cells were aspirated, followed by addition of 100 µL of plant extract solution diluted in RPMI medium (two-fold dilutions, ranging from 0.78–100 µg/mL) and another 100 µL of growth medium (supplemented RPMI) were then added to each well. After incubation at 37°C/5% CO_2_ for a further 3 days, the results were quantified using 3-(4, 5-dimethylthiazol-2-yl)-2, 5-diphenyltetrazolium bromide (MTT, Invitrogen, No: M-6494) as per the manufacturer's instructions. The optical density (OD) was measured at 540 nm using a Bio-Rad iMark TM microplate reader. The percentages of cell viability were based on the amount of living cells in compound-treated cells relative to cell controls (defined as 100% viability). Cytotoxicity graphs were then generated by plotting percentage of cell viability versus concentration of extracts. Using regression analysis of cytotoxicity curves (in Microsoft excel), a trendline that best suited the curve was selected and the corresponding equation was used to calculate 50% cytotoxic concentrations (CC_50_) [29].

### *In vitro* micro-inhibition assay

The activity of plant extracts against influenza viruses was evaluated according to a method described elsewhere [29], albeit some modifications. Briefly, 96-well plates were seeded with 3×10^4^ cells/well and incubated for 24 h at 37°C with 5% CO_2_ until a confluent monolayer was attained. The cells were washed twice with PBS, and two-fold serial dilutions of plant extracts (0.78–100 µg/ml) in RPMI medium were challenged with 100 TCID_50_ of either of the two virus strains. To all wells, 100 µL of RPMI medium supplemented with 2 µg/mL trypsin (virus growth medium) were added. After incubation for three days at 37°C/5% CO_2_, the results were quantified as previously described. The antiviral activity curve was then generated by plotting percentages of virus inhibition against concentrations of extracts. IC_50_, the concentration of extract essential to reduce virus-induced CPE by 50%, was expressed relative to the virus control employing dose-response curves. Using regression analysis of antiviral activity curves (in Microsoft excel), a trendline that best suited the curve was selected and the corresponding equation was used to calculate IC_50_ values [29].

### Time-of-addition assay

The antiviral effects of extracts were evaluated at different times of viral infection as described by Chiang et al. [30]. Briefly, 100 µL/well of each plant extract, serially diluted in RPMI at four concentrations (0.78, 12.5, 25 and 50 µg/mL), were added to 80% confluent MDCK cells at either 1 or 2 hours prior to infection (-1 and -2, respectively), at the time of infection (0), or 1 or 2 hours after viral infection (+1 and +2, respectively). The infection was performed by adding 100 µL/well of either H1N1 or H3N1 (100 TCID_50_). The various time points (−1, −2, 0, +1, +2) were tested independently in separate plates. 100 µL of virus growth medium was added to each well and the plates were then incubated for three days at 37°C/5% CO_2_, after which the virus inhibition was quantified as described earlier.

### Virus binding (attachment) assay

To assess the activity of the compounds in inhibiting viral binding, an attachment assay adapted from De Logu *et al.* [31] was performed. Briefly, 80% confluent cells were chilled at 4°C for 1 hour followed by infection with 50 µL/well of H1N1 or H3N1 (200 TCID_50_) and simultaneous supplementation with 100 µL/well of each plant extract at four concentrations (0.78, 12.5, 25, 50 µg/ml). All plates were held at 4°C for a further 3 h, after which the supernatant was removed; cells were washed twice with ice-cold PBS and the medium was replaced with an equal volume of RPMI and virus growth medium, and incubated for a further three days at 37°C/5% CO_2_. MTT was employed to evaluate cell viability and the percentage of viral inhibition was calculated in relation to the virus control wells.

### Penetration assay

The effect of plant extracts on viral penetration was studied according to a method described elsewhere [32]. Briefly, 80% confluent cells were chilled at 4°C for 1 hour prior to infection with H1N1 or H3N1 (200 TCID_50_) in virus growth medium and held at 4°C for further three hours. After the incubation period, specific concentrations of extracts (0.78, 12.5, 25 or 50 µg/mL) were added in triplicates to the wells with virus. The activity was studied at three time intervals (30, 60 and 120 min) employing one plate per interval at 37°C/5% CO_2_. After the specified time interval, the supernatant was removed and treated with acidic PBS (pH 3) for 1 min to inactivate unpenetrated virus [33], and finally treated with alkaline PBS (pH 11) for neutralization. Cells were washed once with PBS (pH 7.4) and overlaid with an equal volume of RPMI and virus growth media. After three days incubation at 37°C/5% CO_2_, cell viability was evaluated using MTT.

### Neuraminidase (NA) inhibition assay

The NA-Fluor™ Influenza Neuraminidase Assay Kit (Life Technologies, No: 4457091) was employed to test the effects of extracts on the viral neuraminidase of both H1N1 and H3N1 strains as per the manufacturer's instructions. The virus stock was titrated by performing NA activity assay and the optimum virus dilution for the neuraminidase inhibition assay was selected. Two-fold serial dilutions of plant extracts (0.3–25 µg/mL) were tested for NA inhibitory activity. Zanamivir and Oseltamivir were included as positive controls in the assay and tested at nanomolar concentrations (10^−2^ to 10^4^ nM). Fluorescence was measured using a POLARstar Omega fluorescence polarization microplate reader (excitation 355 nm, emission 460 nm). IC_50_ values were determined from dose-response data using a sigmoidal curve-fitting generated and analysed using GraphPad Prism Software.

### Hemagglutination inhibition (HI) test

An HI assay was used to determine the effect of extracts on virus adsorption [34]. Briefly, two fold serial dilutions of the extract (0.78–100 µg/mL) were prepared in PBS and an equal volume (25 µL/well containing 4HAU) of the virus stock was added to each well in a round-bottomed 96-well microtitre plate in triplicate. Subsequently, 50 µL of 5% chicken red blood cells (CRBC) were added to all wells and mixed. The following controls were included in every plate (i) Zanamivir (ii) Oseltamivir, (iii) CRBC without virus, (iv) CRBC with virus devoid of extract and (v) CRBC with extracts devoid of virus (vi) non-commercial anti-HA MAb for H3N1 with an antibody titre of 80 and anti-HA MAb for H1N1 with antibody titre of 200 (provided by Professor Lorena Brown, Department of Microbiology and Immunology, The University of Melbourne), 1∶8 dilution of either of the two antibodies in PBS were included in the assay. The hemagglutination reactions were observed after 30 minutes incubation at room temperature.

### RDE treatment

The effect of Receptor Destroying Enzyme (RDE, Denka Seiken Co. Ltd., Tokyo, Japan) treatment upon the antiviral activity of the extracts was studied through *in vitro* micro inhibition and HI assays. The extracts were added to RDE solutions in the ratio 1∶3 and incubated at 37°C for 20 hours according to the manufacturer's recommendations. The purpose of this experiment was to eliminate compounds that may possess sialic acid-like structures which mimic the receptors of RBC and compete for hemagglutinin [35] _ENREF_25. The extract and RDE mixture was inactivated at 56°C for 60 minutes and then subjected to the assays.

### Trypsin treatment

The effect of trypsin (Sigma, No: T1426) upon the antiviral activity of the extracts was studied through an *in vitro* micro inhibition assay and HI assays. The plant extracts were treated with 4 µL of trypsin (1 mg/mL in 1% acetic acid), incubated at 37°C for 24 hours and followed by incubation at 56°C for 60 minutes before performing the assays. Plant extracts subjected to the same temperature without trypsin and extracts that were neither subjected to temperature nor trypsin treatments were included as controls.

### Statistical analysis

All treatments were performed in triplicates and each experiment was independently repeated at least twice. The data were expressed as mean ± standard error of the mean (SEM). The results of the antiviral activity assays were analysed with a one-way ANOVA test and a significance level (*p* value) of 0.05 or 0.01 was considered to compare the means.

## Results

### Cytotoxicity studies of plant extracts

**Figure 1:**
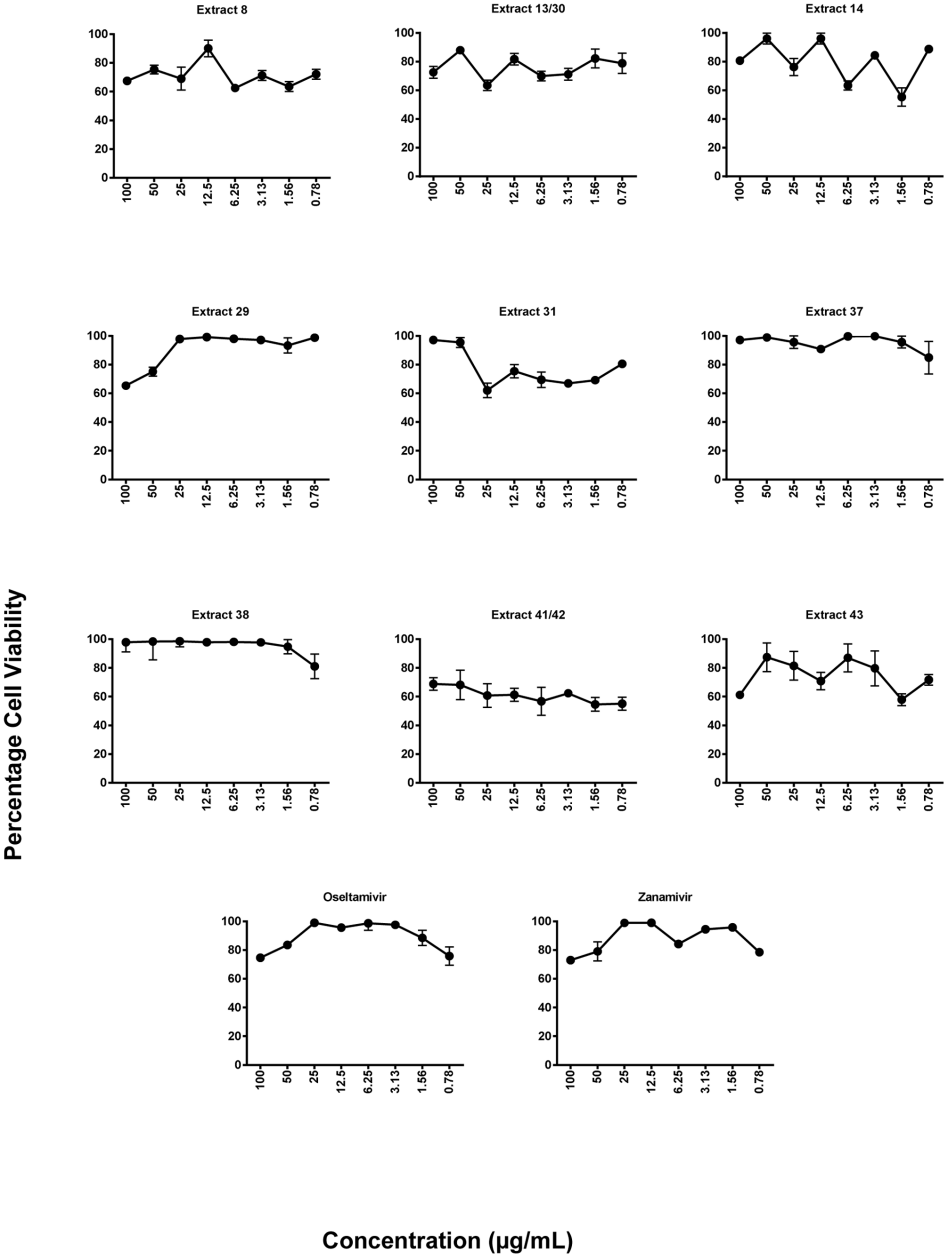
Cytotoxicity effects of plant extracts. Following overnight incubation of cells seeded at 4×10^3^ cells per well into 96-well flat-bottomed microtitre plates, the media were aspirated and overlaid with 100 µL of two-fold serial dilutions of plant extract (0.78–100 µg/mL) with an additional 100 µL of growth medium (supplemented RPMI). After three days incubation, cell viability was evaluated using MTT and percentage cell viability calculated relative to cell control wells. Representatives of two independent experiments performed in triplicate are shown. Statistical analysis showed that data were significant with *p*<0.05 (one way ANOVA).

The medicinal plant extracts were screened for cellular toxicity in order to determine appropriate concentrations for the *in vitro* micro inhibition assays. As detailed in Table 1, there were duplicate samples among the plant extracts; extracts 13 and 30 were obtained from two sources of the same species collected in the same location at different times while extracts 41 and 42 were obtained from different parts of the same plant. As shown in Table 2, the concentration associated with 50% cytotoxicity (CC_50_), estimated using regression analysis, was greater than the highest tested concentration (>100 µg/mL). In this case, the CC_50_ was an estimated theoretical value obtained by extrapolation of the results in Figure 1. Though most of the plant extracts demonstrated minimal cytotoxicity at concentrations less than 6.25 µg/mL compared to the cell control wells, serial dilutions ranging from 0.78–100 µg/mL were chosen for *in vitro* micro-inhibition assays, since the toxicity demonstrated by the plant extracts were similar to Zanamivir and Oseltamivir (Table 2).

**Table 1:** Medicinal plant extracts from Sarawak demonstrating antiviral activity against H3N1 and H1N1 strains.

No	Voucher specimen no.	Plant part	Botanical name (Family)	Medicinal use*
8	SABC 0782	Whole plant	*Mussaenda elmeri* (Rubiaceae)	Conjunctivitis, headache
13	SABC 1753	Leaves	*Trigonopleura malayana* (Euphorbiaceae)	Cough
14	SABC 1768	Whole plant	*Santiria apiculata* (Burseraceae)	Flu, headache
29	SABC 1984	Stems	*Anisophyllea disticha* (Anisophylleaceae)	Fever
30	SABC 1996	Leaves	*Trigonopleura malayana* (Euphorbiaceae)	Cough
31	SABC 1988	Roots	*Trivalvaria macrophylla* (Annonaceae)	Flu, headache
37	SABC 3970	Stems	*Baccaurea angulata* (Euphorbiaceae)	Conjunctivitis
38	SABC 3809	Leaves	*Tetracera macrophylla* (Dilleniaceae)	Cough
41	SABC 1528	Whole plant	*Calophyllum lanigerum* (Clusiaceae)	Potential to treat AIDS
42	SABC 1528	Stems	*Calophyllum lanigerum* (Clusiaceae)	Potential to treat AIDS
43	SABC 4492	Stems	*Albizia corniculata* (Fabaceae)	Sore throat

* Information derived from; Chai 2006 [50], Salleh 2002 [51], Yaacob 2009 [52], Maji 2010 [53], Focho 2010 [54].

**Table 2:** Cellular toxicity and inhibitory concentration of anti-influenza extracts against H3N1 and H1N1 strains.

Extract	CC_50_^a^ (µg/mL)	IC_50_^b^ (µg/mL) against H3N1	IC_50_^b^ (µg/mL) against H1N1
8	133.0±14.2	19.4±4.5	<0.78
13/30	130.0±3.5	15.5±2.8/6.8±0.2	17.0±0.3/13.1±0.5
14	136.3±8.2	7.3±2.7	9.3±1.5
29	157.2±9.6	39.3±6.9	37.5±1.5
31	106.7±2.1	15.6±1.5	6.3±2.0
37	149.0±7.1	27.2±1.6	17.6±0.6
38	165.0±10.0	14.3±1.7	2.8±1.8
41/42	140.7±0.9	10.8±3.6/2.0±1.3	6.3±2.4/9.9±0.2
43	109.8±10.1	6.7±0.5	1.5±0.7
Zanamivir	124.8±4.9	<0.78	<0.78
Oseltamivir	123.0±2.6	<0.78	<0.78

CC_50_^a^ represents the concentration of plant extract required to reduce the number of viable cells by 50% relative to control wells without test compound, calculated from dose–response data, CC_50_ value estimated using regression analysis was greater than the highest tested concentration (>100 µg/mL). In this case, the result was an estimated theoretical value obtained by extrapolation of the results in Figure 1.

IC_50_^b^ represents the concentration of plant extract needed to reduce the viral inhibition by 50% relative to virus control wells without test compound, calculated from dose–response data of virus inhibition. Plant extracts (0.78–100 µg/mL) in RPMI medium were challenged with 100 TCID_50_ of either of the two virus strains.

### Inhibitory effects of plant extracts on influenza virus

**Figure 2:**
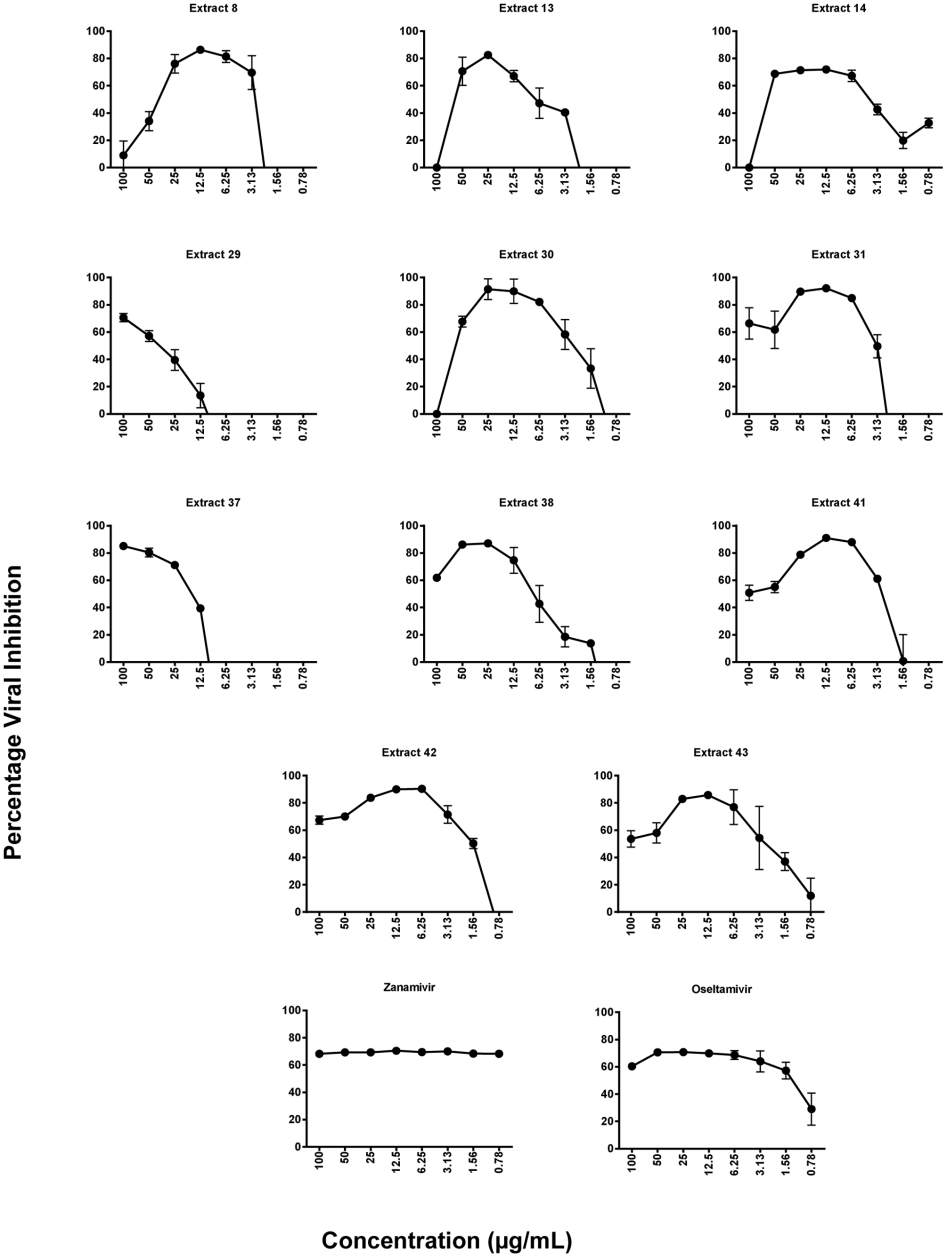
Inhibitory effects of plant extracts on H3N1 influenza virus. Cells at 80% confluency were treated with two-fold serial dilutions of plant extract (0.78–100 µg/mL) and 100 TCID_50_ of H3N1 simultaneously. All wells were provided with 100 µL of RPMI medium supplemented with 2 µg/mL trypsin (virus growth medium). Cell viability was evaluated using MTT and viral inhibition percentage calculated relative to virus control wells. Representatives of two independent experiments performed in triplicate are shown. Statistical analysis showed that data were significant with *p*<0.05 (one way ANOVA).

**Figure 3:**
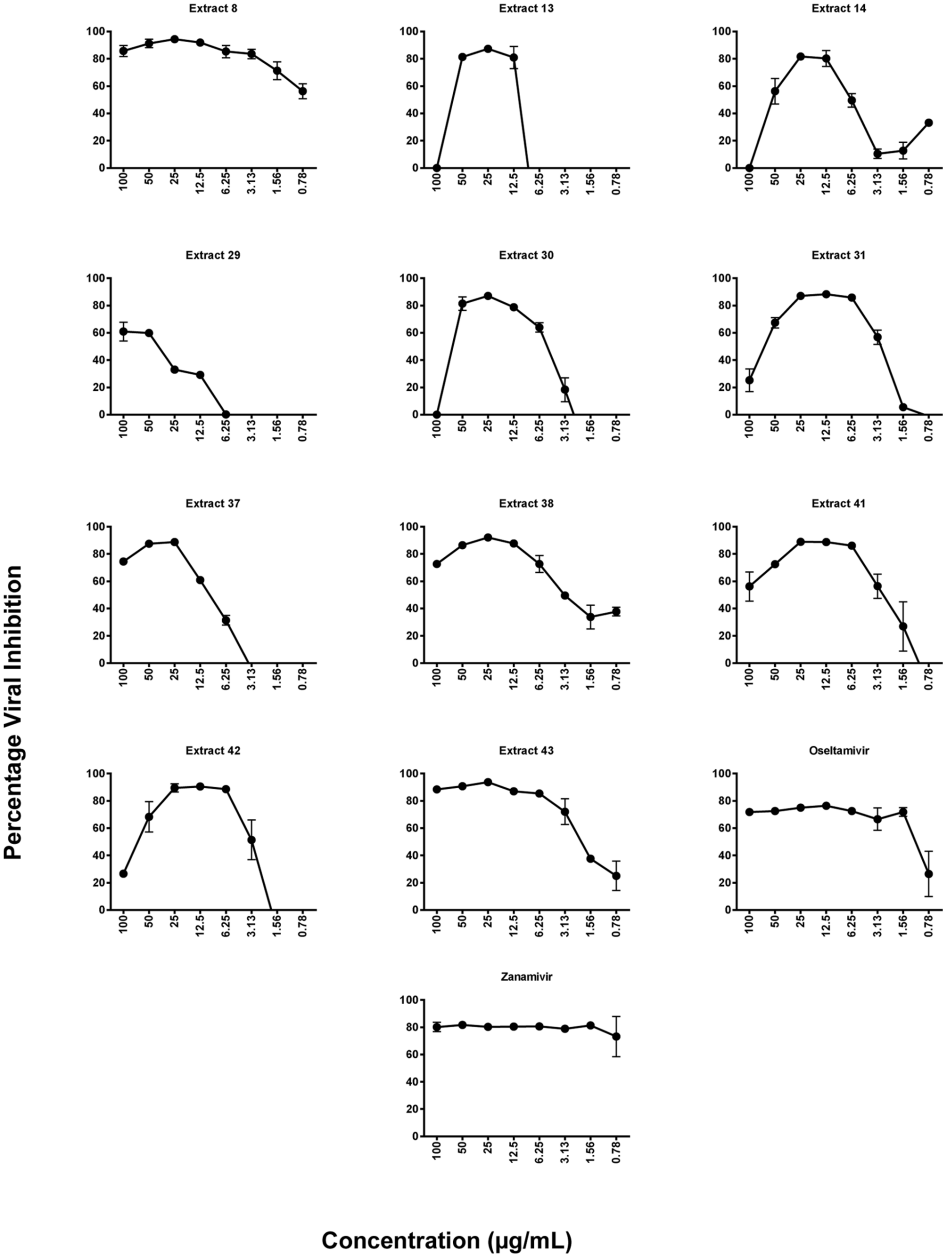
Inhibitory effects of plant extracts on H1N1 influenza virus. Cells at 80% confluency were treated with two-fold serial dilutions of plant extract (0.78–100 µg/mL) and 100 TCID_50_ of H1N1 simultaneously. All wells were provided with 100 µL of RPMI medium supplemented with 2 µg/mL trypsin (virus growth medium). Cell viability was evaluated using MTT and viral inhibition percentage calculated relative to virus control wells. Representatives of two independent experiments performed in triplicate are shown. Statistical analysis showed that data were significant with *p*<0.05 (one way ANOVA).

The plant extracts were subjected to a high throughput *in vitro* micro-inhibition screening assay to determine antiviral activity. Those demonstrating more than 50% viral inhibition were deemed to have anti-influenza activity. A number of plant extracts exhibited inhibitory activity against influenza virus strain Mem-Bel (H3N1). However, only eleven extracts consistently reduced viral infectivity by greater than 50% (Table 2). The same eleven extracts also mediated significant antiviral activity against the PR8 (H1N1) strain. The antiviral activity curves for all the extracts against H3N1 and H1N1 are shown in Figures 2 and 3, respectively. Duplicate hits in the assay (extracts 13 and 30, and extracts 41 and 42) confirmed the consistency of this screening procedure. The plant extracts were most active at 12.5–50 µg/mL with the exception of extract 29, which was active between 50–100 µg/mL of extract.

### Anti-influenza extracts studied with a time-of-addition assay

The antiviral potentials of the eleven active extracts were tested against H3N1 and H1N1 strains at different times (−1 h, −2 h, 0 h, +1 h, and +2 h) relative to virus inoculation. All extracts inhibited the viruses by more than 50% at all time points tested, except for the -2 h time for extract 14 against the H1N1 strain; 12.5–50 µg/mL was most active in virus inhibition (data not shown).

### Inhibitory effects of anti-influenza extracts on the attachment of H3N1 and H1N1

**Figure 4:**
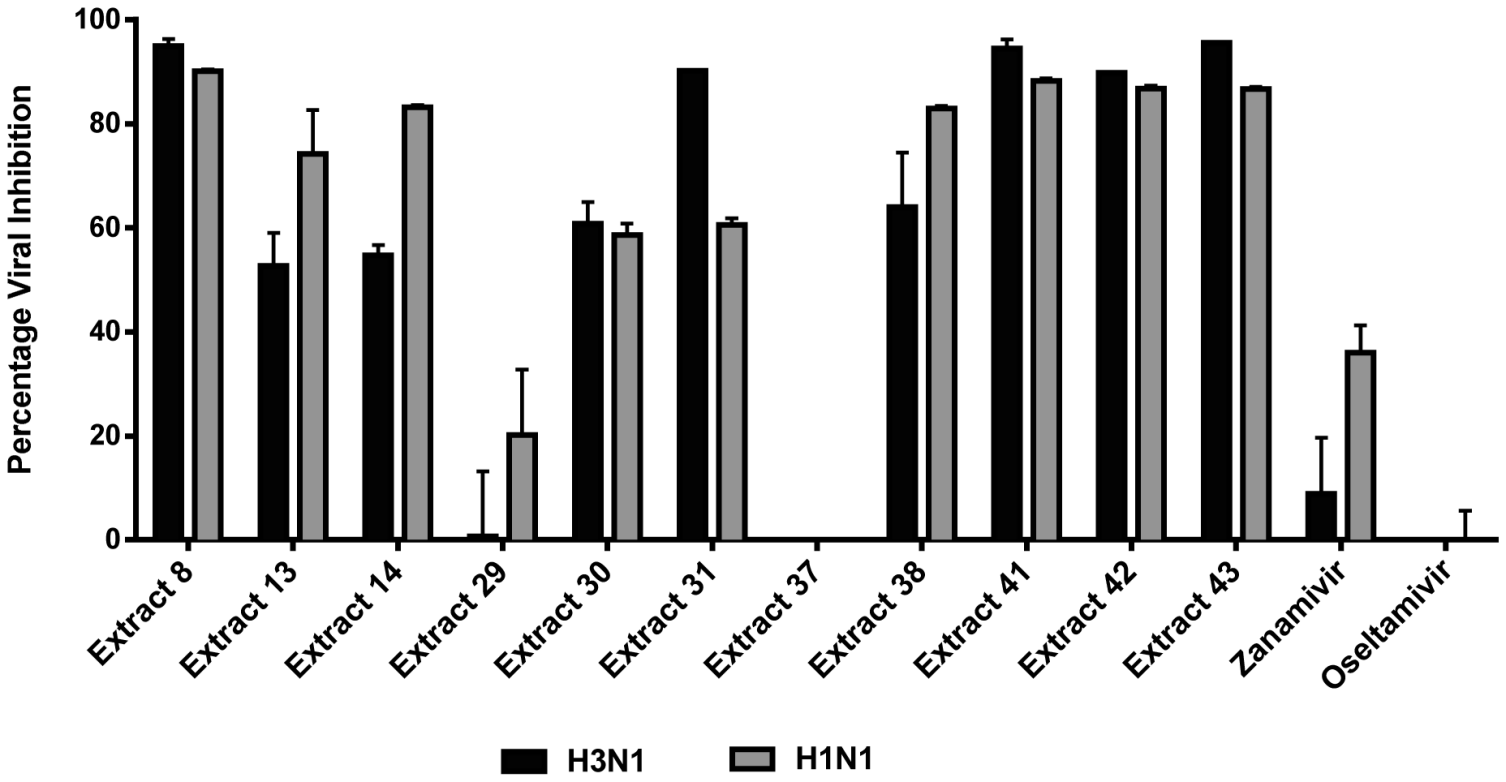
Inhibitory effects of plant extracts on the binding of H3N1 and H1N1 virus to MDCK cells. Cells at 80% confluency and pre-chilled at 4°C for an hour were infected with 200 TCID_50_ of H1N1 or H3N1 followed by supplementation with plant extract at 25 µg/mL concentration. After 3 h incubation at 4°C, cells were washed twice with ice-cold PBS and overlaid with RPMI and virus growth medium. Cell viability was evaluated using MTT and viral inhibition percentage calculated relative to virus control wells. Effect of plant extracts on virus binding at a concentration of 25 µg/mL is shown. Representatives of two independent experiments performed in triplicate are shown. Statistical analysis showed that data were significant with *p*<0.05 (one way ANOVA).

Plant extracts were tested for their ability to inhibit viral attachment using a virus binding assay. As shown in Figure 4, nine out of eleven extracts demonstrated more than 50% viral inhibition against both H3N1 and H1N1 strains at 25 µg/mL. The results support HI activity demonstrated by extracts 8, 41, 42 and 43. Despite lacking HI activity, extracts 13, 14, 30, 31 and 38 demonstrated significant inhibitory effects on the binding of influenza virus. As expected, the established neuraminidase inhibitors (NAI), Zanamivir and Oseltamivir, did not inhibit virus binding. As shown in Table 3, plant extracts inhibited the binding of H3N1 and H1N1 viruses depending on the concentration of plant extracts used in the assay. The virus inhibition percentages of the wells that received higher concentrations of the plant extracts (50 or 12.5 µg/mL) were greater than the wells that were treated with lesser concentrations of the extract (0.78 µg/mL), with the exception of extract 14, which was inactive in inhibiting the binding of influenza virus at 50 µg/mL but exhibited inhibitory activity at 0.78 µg/mL against H3N1. Extract 42 was also shown to inhibit the binding of H3N1strain at 0.78 µg/mL, at which other extracts, except extract 14 were inactive.

**Table 3:** Inhibitory effects of anti-influenza extracts on the binding of H3N1 and H1N1 strains.

Extract	Percentage viral inhibition against the binding of H3N1 strain			Percentage viral inhibition against the binding of H1N1 strain		
	Concentration (µg/mL)			Concentration (µg/mL)		
	50	12.5	0.78	50	12.5	0.78
8	92.7±0.8	95.5±0.9	-	88.8±1.0	89.3±0.7	-
13	88.8±2.5	56.1±8.5	-	89.5±0.1	56.4±1.6	-
14	-	69.2±1.3	65.0±0.3	-	89.3±3.7	-
29	-	-	-	-	-	-
30	88.3±2.4	62.8±1.1	-	76.0±3.0	-	-
31	91.0±0.1	90.4±0.7	-	62.4±1.0	61.9±1.0	-
37	-	-	-	-	-	-
38	69.2±4.5	63.2±3.5	-	79.0±3.0	82.6±0.43	-
41	95.4±0.1	87.9±2.0	-	89.7±1.3	88.1±0.2	-
42	90.3±0.3	90.1±0.2	57.9±6.5	86.8±0.2	88.1±0.6	-
43	93.5±0.1	93.0±2.8	-	83.0±1.5	87.2±0.4	-
Zanamivir	-	-	-	-	-	-
Oseltamivir	-	-	-	-	-	-

The activity of extracts against the binding of influenza virus at a concentration of 50, 12.5 and 0.78 µg/mL is shown along with standard errors. Plant extracts were challenged with 200 TCID_50_ of either of the two virus strains. A negative sign indicates lack of anti-influenza activity.

### Inhibitory effects of anti-influenza extracts on the penetration of influenza virus

**Figure 5:**
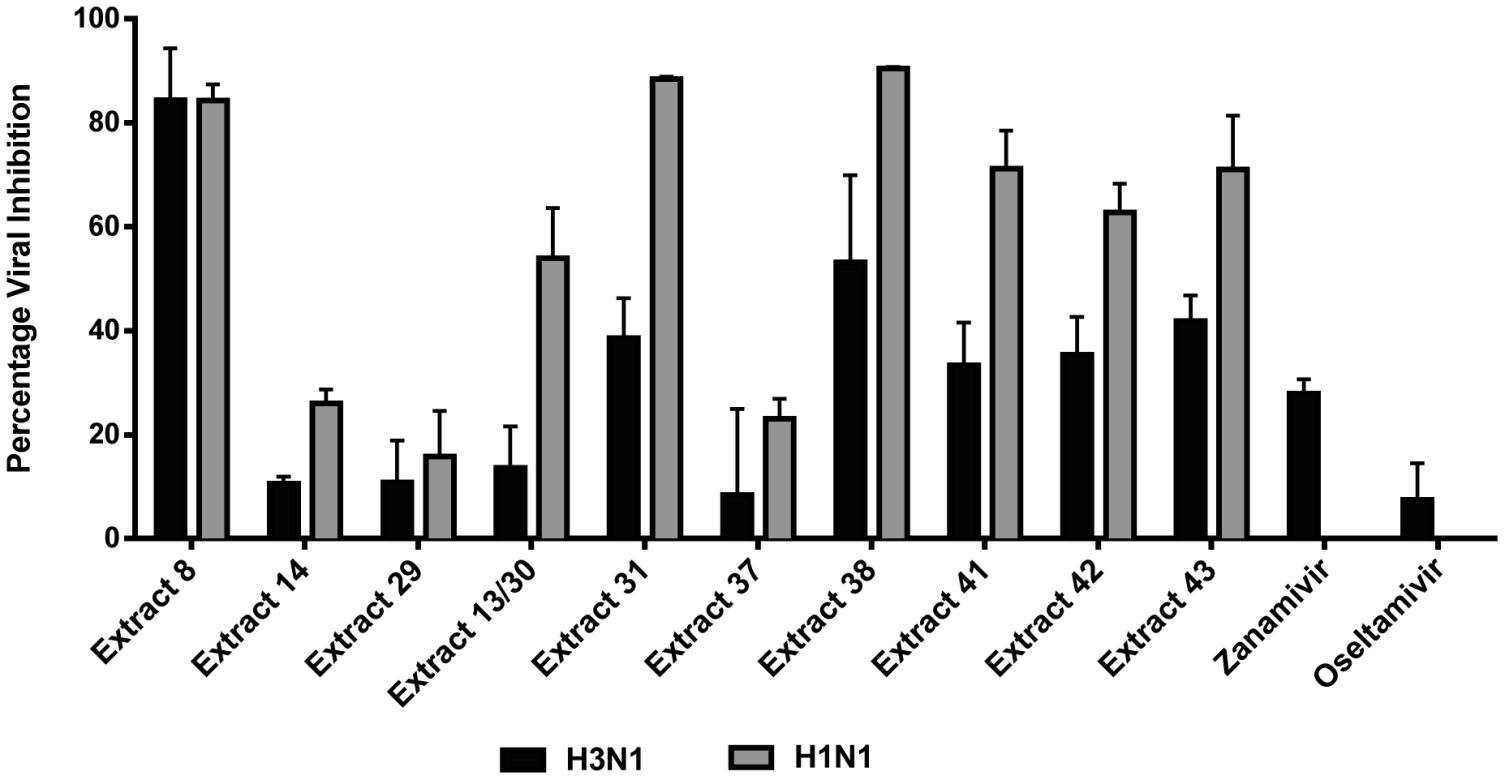
Antiviral activity of plant extracts against the penetration of H3N1 and H1N1 virus at 60 min. Monolayers of MDCK cells (80% confluent) were chilled at 4°C for an hour and then incubated with 200 TCID_50_ of H3N1 or H1N1 viruses at 4°C for 3 h. Plant extracts (25 µg/mL in RPMI medium) were then added in triplicate and incubated for 60 minutes at 37°C/5% CO_2_. Following inactivation and neutralization of unpenetrated virus using acidic and alkaline PBS, respectively, cells were washed with PBS and overlaid with RPMI medium and virus growth medium in equal proportion. Cell viability was evaluated using MTT after three days of incubation at 37°C/5% CO_2_. Data shown are representative of two independent experiments performed in triplicate. Statistical analysis showed that data were significant with *p*<0.05 (one way ANOVA).

As shown in Table 4, all extracts were able to prevent viral penetration, with the exception of extract 29 which was ineffective at all time points and extract 37 which was active only against H3N1 strain. These data support HI results obtained using extracts 8, 41, 42 and 43. Figure 5 shows the effects of 25 µg/mL extracts against H3N1 and H1N1 strains at 60 min. Four plant extracts (8, 30, 31 and 38) demonstrated virus inhibition at all three time points, including the effect of 12.5 and 50 µg/mL of plant extracts at 60 min (data not shown). For three HI extracts (41, 42 and 43), inhibition of virus penetration increased over time as the antiviral activity of the extract at 60 and 120 min was greater than that observed at 30 min against H3N1 strain. It is noteworthy that some extracts lacking HI activity (13/30, 14, 31, 37 and 38) were shown to inhibit virus penetration. Although Zanamivir and Oseltamivir should normally act against virus release and not virus penetration, surprisingly, 50 µg/mL Zanamivir showed 70% viral inhibition of H3N1 after 60 min (data not shown). As expected, Oseltamivir was inactive against both viruses in the assay.

**Table 4:** Inhibitory effects of anti-influenza extracts on the penetration of H3N1 and H1N1 strains at 30 and 120 min.

Extract	Antiviral activity of extract (concentration in µg/mL) against the penetration of H3N1 strain				Antiviral activity of extract (concentration in µg/mL) against the penetration of H1N1 strain			
	30 min		120 min		30 min		120 min	
	50	25	50	25	50	25	50	25
8	81.7±0.7	-	90.4±3.6	85.3±7.3	89.8±3.3	80.5±9.7	90.1±0.8	89.5±0.1
13/30	60.7±1.1	-	63.5±4.5	-	76.8±1.7	67.9±8.7	85.6±1.9	64.6±2.5
14	85.7±0.5	64.2±1.2	71.8±0.8	64.1±1.6	53.8±1.9	-	-	-
29	-	-	-	-	-	-	-	-
31	92.8±0.3	81.8±9.2	78.3±2.6	56.1±14.1	58.2±3.2	65.2±7.9	86.7±1.5	87.4±0.6
37	73.7±1.9	-	59.4±2.4	-	-	-	-	-
38	88.2±4.8	65.0±2.7	51.4±1.4	52.7±3.1	54.2±9.6	64.5±1.8	82.2±4.6	91.8±0.6
41	73.4±5.2	-	91.9±1.8	68.7±16	79.9±1.1	73.5±12.2	79.8±0.9	79.3±0.3
42	83.7±2.3	58.0±7.7	85.8±2.7	65.8±0.8	57.1±6.2	-	60.9±7.6	54.0±1.8
43	76.0±9.0	-	86.5±1.2	91.2±1.0	71.3±1.0	68.2±1.8	-	65.4±0.3
Zanamivir	-	-	-	-	-	-	-	-
Oseltamivir	-	-	-	-	-	-	-	-

The activity of extracts against the penetration of influenza virus (200 TCID_50_) at a concentration of 50 and 25 µg/mL is shown along with standard errors. Threshold concentration of active components that prevent virus penetration appears between 25–50 µg/mL of plant extracts. A negative sign indicates lack of anti-influenza activity.

### NA inhibitory effects of plant extracts

The influenza virus NA glycoprotein has sialidase activity and mediates the release of viral progeny from the infected cell, thus promoting virus transmission and spread [36]. In addition, the viral NA removes sialic acid from glycans expressed by the viral HA glycoprotein, thereby preventing self-aggregation of virions [37]. All eleven extracts were tested for NA inhibitory activity against Mem-Bel (H3N1) and PR8 (H1N1) using the NA-Fluor™ Influenza Neuraminidase Assay Kit. Increasing concentrations of plant extracts were associated with decreased relative fluorescence (Table 5), consistent with inhibition of NA activity. IC_50_ values indicated that extracts 8 and 43 reduced NA activity at a lower concentration than the other extracts. It should be noted that we have tested crude plant extracts, thus the results cannot be directly compared with Zanamivir and Oseltamivir, as these commercially available drugs were tested at nanomolar concentrations.

**Table 5:** Neuraminidase inhibitory activity of anti-influenza extracts.

Extract	IC_50_ (µg/mL) against H3N1	IC_50_ (µg/mL) against H1N1
8	1.15±0.03	0.59±0.10
14	7.81±0.81	4.33±0.12
29	4.19±1.26	4.57±0.35
13/30	3.87±0.07	2.46±0.47
31	5.51±0.21	2.21±0.18
37	6.70±1.35	9.91±0.30
38	3.12±0.04	0.52±0.11
41/42	4.47±0.15/6.35±0.87	5.76±0.05/5.34±0.11
43	0.43±0.01	1.38±0.07
Antiviral drug	IC_50_(nM) against H3N1	IC_50_(nM) against H1N1
Zanamivir	6.0±0.58	7.12±0.80
Oseltamivir	6.90±3.1	16.10±0.30

The plant extracts' neuraminidase inhibitory activity was measured at concentration ranging between 0.3 to 25 µg/mL whereas the controls Zanamivir and Oseltamivir were assayed at 0.01 to 10,000 nM as recommended by the manufacturer. The optimum virus dilution for the neuraminidase inhibition assay was selected by titration of virus stock in an NA activity assay; 1:8 dilution of either of the virus was selected in the NA activity assay to perform NAI assay.

### Inhibitory effects of plant extracts on influenza virus-induced hemagglutination

**Figure 6:**
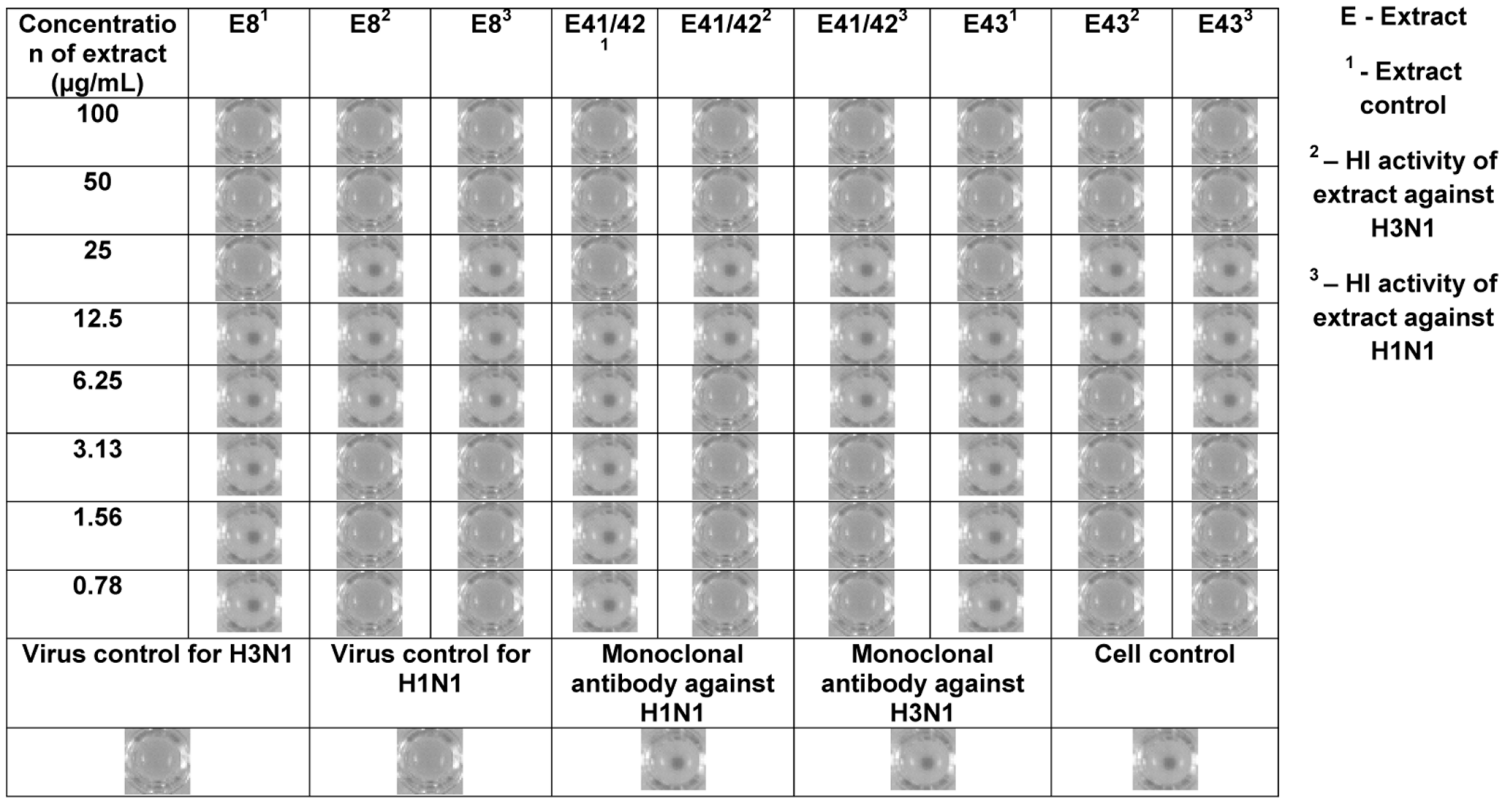
Inhibitory effects of plant extracts on the hemagglutination of H3N1 and H1N1 viral strains. HI activities of four extracts (0.78–100 µg/mL) against 4HAU/25 µL of virus are shown. The following controls were included on each plate; (i) extract controls with extract and chicken red blood cells (CRBC) only, (ii) virus controls containing virus and CRBC and (iii) cell controls containing only CRBC. Monoclonal antibody against the HA of either H3N1 or H1N1 strains were included as a positive control. The antibody titres for monoclonal antibody against H3N1 and H1N1 were 80 and 200, respectively; 1:8 dilution of either of the two antibodies in PBS were employed in the assay. Data are shown from one of three independent experiments, each performed in triplicate.

The influenza virus HA mediates attachment to the sialic acid residues expressed by the glycoproteins and glycolipids of host cells, which is a critical step in the initiation of infection [38]. Similarly, the viral HA binds to sialic acids expressed on the surface of erythrocytes resulting in hemagglutination. Thus, we examined the ability of plant extracts to inhibit virus-induced hemagglutination using a hemagglutination inhibition (HI) assay. As shown in Figure 6, four out of eleven extracts mediated HI activity against Mem-Bel and PR8 viruses at specific concentrations. Extract controls were included to study the direct effect of extracts on chicken red blood cells in the absence of Mem-Bel and PR8 viruses. All four HI extracts exhibited hemolysis above 25 µg/mL in the absence of viruses. Some hemolytic concentrations of extract control resulted in HI when the virus was included, suggesting that extract components preferentially attach to the virus rather than to the erythrocytes. Plant extracts that mediated HI activity against Mem-Bel and PR8 strains were active in preventing virus-induced hemagglutination at concentrations ranging between 12.5-25 µg/mL.

### Effect of RDE treatment on the antiviral activity of extracts

**Figure 7:**
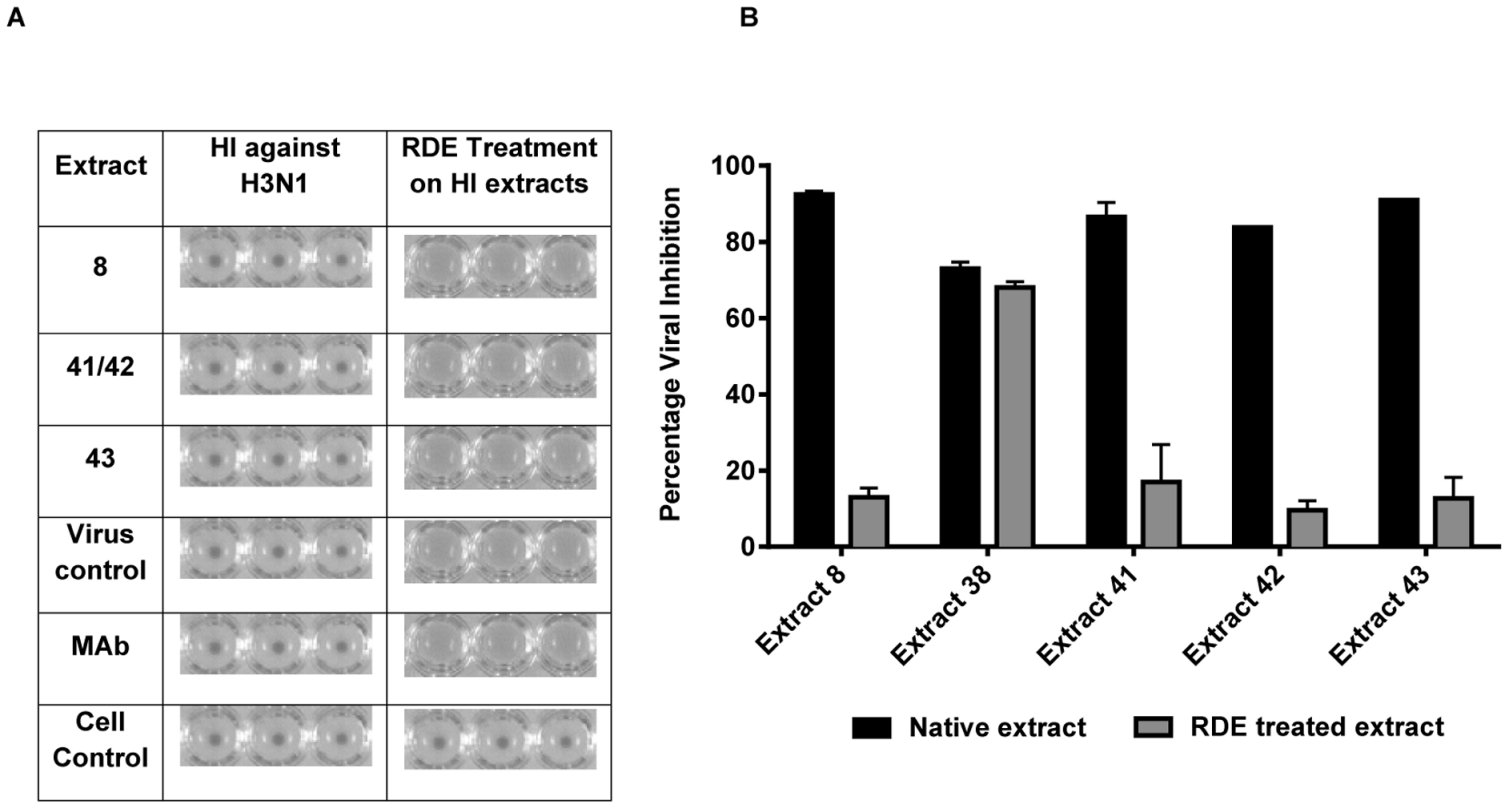
Effect of RDE treatment on the antiviral activity of plant extracts. A. Inhibitory effect of plant extracts on the hemagglutination of H3N1 viral strain. HI activities of four extracts (25 µg/mL) treated with RDE against 4HAU/25 µL of virus are shown. (i) Virus controls containing virus and CRBC and (ii) cell controls receiving CRBC only are shown. Corresponding RDE treated monoclonal antibody which acts against the HA of H3N1and extracts that mediate HI activity without RDE treatment were included in all plates as positive controls. The experiment was performed in triplicate. B. Loss of efficacy in antiviral inhibition of HI extracts against H3N1 strain. An *in vitro* micro-inhibition assay was used to assess the ability of plant extracts to inhibit H3N1 (100 TCID_50_) influenza virus. Extracts were either treated with RDE as per the manufacturer's instructions or left in their native form without RDE treatment. Data shown are representative of two independent experiments performed in triplicate. Statistical analysis showed that data were significant with *p*<0.05 (one way ANOVA).

Four extracts which were shown to interfere with hemagglutination of chicken red blood cells were treated with RDE in order to eliminate compounds that might contain sialic acid mimics that compete with the RBC receptors for virus hemagglutinin. An HI assay was performed with RDE-treated extracts which were originally able to prevent hemagglutination. As shown in Figure 7, RDE treatment removed HI activity originally exhibited by all four extracts. Further, an *in vitro* micro-inhibition assay was performed with the RDE-treated extracts against H3N1 and H1N1 viral strains. As shown in Figure 7, there was a significant reduction in the antiviral efficacy of extracts with HI potential whereas non-HI extract 38, included as a negative control, did not show any significant difference in viral inhibition before and after RDE treatment. A similar pattern in the percentage viral inhibition was observed with the H1N1 strain exposed to RDE-treated extracts (data not shown).

### Effect of Trypsin treatment on the antiviral activity of extracts

**Figure 8:**
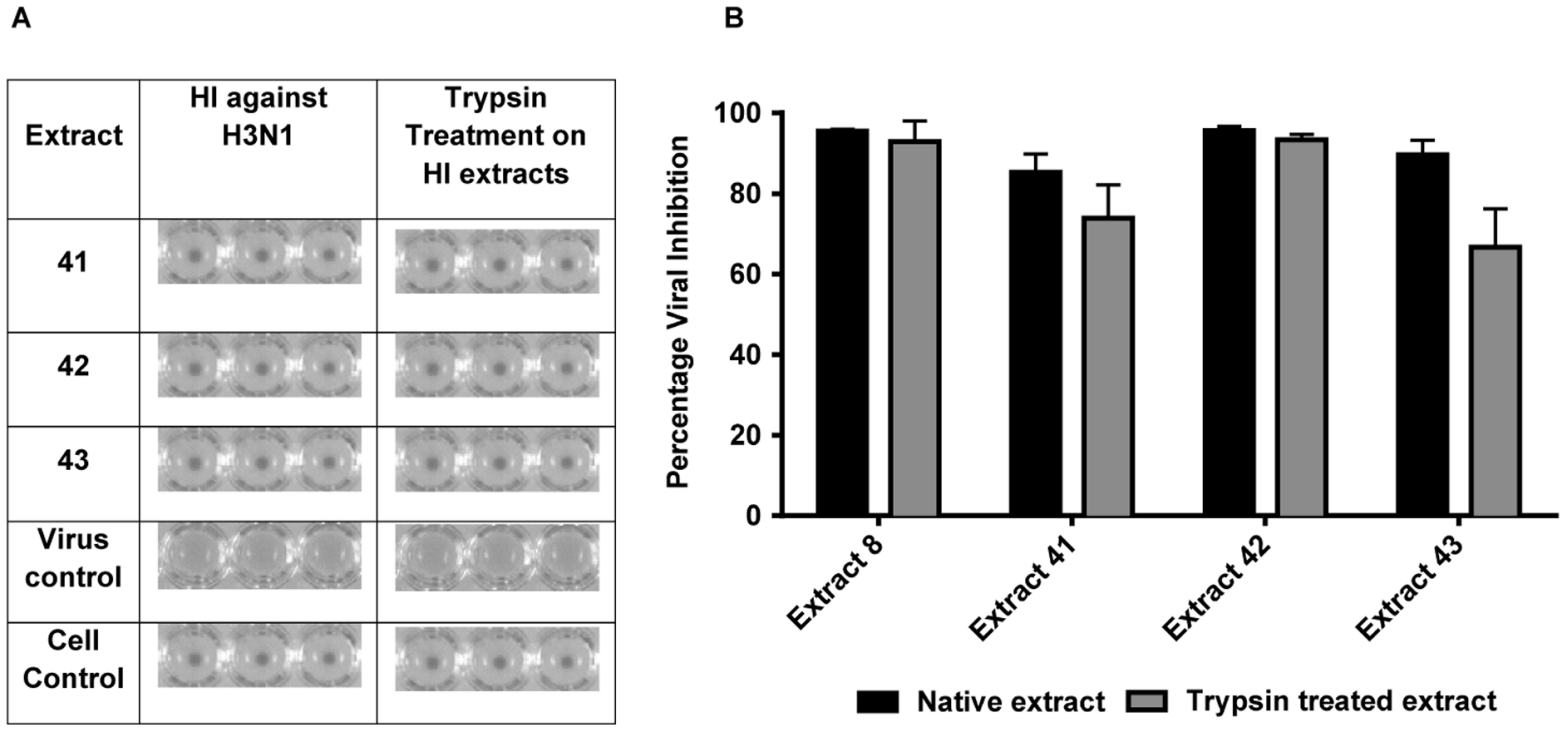
Effect of trypsin treatment on the antiviral activity of plant extracts. A. Inhibitory effect of plant extracts on the hemagglutination of H3N1 viral strain. HI activities of three extracts (50 µg/mL) treated with trypsin against 4HAU/25 µL of virus are shown. (i) Virus controls containing virus and CRBC and (ii) cell controls receiving CRBC only are shown. Extracts that mediate HI activity without trypsin treatment were included in all plates as positive controls. The experiment was performed in triplicate. B. Antiviral inhibition of HI extracts against H3N1 strain. An *in vitro* micro-inhibition assay was used to assess the ability of plant extracts to inhibit H3N1 (100 TCID_50_) influenza virus. Extracts (3.13–100 µg/mL) were either treated with trypsin for 24 hours at 37°C, followed by incubation at 56°C for 60 minutes or subjected to temperature without trypsin. Activity of extracts at 50 µg/mL concentration is shown in the figure. Data shown are representative of two independent experiments performed in triplicate. Statistical analysis showed that data were significant with *p*<0.05 (one way ANOVA).

Plant extracts that were shown to prevent virus induced haemagglutination were treated with trypsin in order to denature any protein that might be the cause of such inhibition. An *in vitro* micro-inhibition assay was initially performed to determine the activity of trypsin-treated extracts and controls against H3N1 and H1N1 strains. As shown in Figure 8, the antiviral activity of plant extracts against the H3N1 virus was not altered by either trypsin treatment or temperature (without trypsin). The trypsin-treated plant extracts and controls were then subjected to an HI assay. As shown in Figure 8, HI activity of plant extracts were exhibited against H3N1 despite trypsin treatment or temperature change. A similar pattern in the percentage viral inhibition and HI activity was observed with the H1N1 strain exposed to trypsin-treated extracts (data not shown).

## Discussion

Phytomedicines have been used since ancient times to treat various infections but clinical studies are limited [39]. In this study, we have identified a number of traditional medicinal plant extracts collected from Sarawak, Malaysia, which displayed anti-influenza activity. Potentially, there are many compounds within any given extract that might mediate antiviral activity. The antiviral compounds present in these extracts may act alone or work in a synergistic manner. The efficacy of several plant extracts used in herbal medicine is directly related to the synergistic effects of bioactive components and derivatives [40].

Safety is a major requirement for an antiviral agent and in the search for new drugs it is important to consider possible secondary effects. The minimal cytotoxicity observed in the extracts investigated may be due to the presence of cytoprotective components. This cytoprotective role of plants have been reported in other studies on plant extracts [41]. In our study, this is an indication that these extracts might serve as potential candidates for the development of safe and less toxic drugs. In general, compounds that are linked to ethnomedical uses are considered to be safe and more effective than substances that lack this framework [14]. Being pure drugs, Zanamivir and Oseltamivir inhibited influenza virus at all concentrations tested (0.78 to 100 µg/mL). Despite the difference in concentration, the anti-influenza drugs followed concentration-independent virus inhibition. Chemical characterization of the active components present in the plant extracts may lead to concentration-independent virus inhibition like that of Zanamivir and Oseltamivir. The amounts of active component(s) present in the plant extracts and their efficiency in preventing virus inhibition play a major role in demonstrating antiviral activity.

All plant extracts tested were shown to exhibit NAI activity. Extracts 8, 38 and 43 demonstrated the lowest IC_50_ range, indicating a higher amount of NAI in the given plant extract or the presence of potent NAI component(s) that may be active even at a lower concentration. Extracts were tested at µg/mL concentrations, unlike Zanamivir and Oseltamivir, which were tested at nanomolar levels. Selection of µg/mL concentration range in NAI assay relies on the likelihood that anti-influenza activity may not be detected at very low concentrations as none of the plant extracts showed activity at concentrations less than 3.13 µg/mL in the *in vitro* micro-inhibition assays. As suggested in the literature, the chemical components in the extracts might have combined with the viral membrane to modify the physical properties of viral neuraminidase [42]. Time of addition assay results also suggest the extracts have NAI activity, since +1 h and +2 h post-infection experiments showed more than 50% viral inhibition. Therefore, the dose and time required for the extract to prevent viral growth are critical and different for each.

Since drug-resistant viruses appear frequently, it is important to identify drugs with a different mode of action to the one observed with the conventional drugs currently used (NAI and adamantanes). A sialylated molecule that can block virus attachment to cellular receptors might act to limit the initial stages of virus infection, compared to NA inhibition that is believed to act largely through preventing release of new virions from virus-infected cells. Moreover, NAI must be administered in the early stages of infection as they are less efficient during the later phases [43]. Four of the extracts mediated HI activity within a specific range of concentrations. It has been reported elsewhere that in HI assays some plant extracts can cause hemolysis at higher concentrations [44].

Hemolysis caused by the extracts in the controls may be attributed to the presence of other compounds apart from those with anti-influenza activity. It is also possible that more than one anti-influenza component may be present in extracts that showed multiple modes of action. The stable interaction between HA and NA, which is vital for the effective entry and release of the virus, may have been disrupted by the anti-influenza component(s) present in the plant extracts. This could also be considered a new anti-influenza pathway as dual action drugs have not previously been used.

The inhibitory effects demonstrated against viral binding and penetration further suggested the HI mode of action of extracts 8, 41, 42 and 43. Interestingly, non-HI extracts 13, 14, 30, 31, 37 and 38 which exhibited NAI activity showed significant inhibition in the viral binding and penetration assays. The major functions of the sialidase activity of influenza NA are to facilitate the release of viral progeny from infected cells and enable viral spread, but NA is also important for viral entry [44]–[46]. The role of NA in removing sialic acid residues from HA could improve fusion and infectivity of influenza virus following three mechanisms:

- Glycoconjugates carried by various lipids and proteins expressed at the surface of the host cell could interfere with the receptor binding site of HA spikes. By masking these glycoconjugates, NA enables the binding of HA to sialic acid receptors present at the host cell surface
- Desialylation of HA by NA could also remove acid sialic residues of HA protein, this partial unmasking could facilitate further binding of HA to the target cell surface receptors, thereby augmenting HA-mediated fusion
- Desialylation of HA could help in the proteolytic cleavage of the HA0 precursor into its functional subunits HA1 and HA2, as indicated by observations where the removal of N-glycosylation sites near HA0 cleavage site could modify its approach to cellular proteases [46]_ENREF_36.

Thus neuraminidase also affects viral entry according to the above-mentioned phenomena. The significant effects exhibited by non-HI extracts in viral binding and penetration could be due to the neuraminidase inhibitory component, which might have played a role in inhibiting viral entry. Similar results have been obtained in previous studies [44]. The inhibitory effect of Zanamivir at the 60 minutes time point in the penetration assay might have also resulted from the NAI pathway.

The loss of HI activity of the extracts following RDE treatment suggests that the responsible components may possess sialic acid-like structures that mimic the receptors of CRBC, thereby competing for viral hemagglutinin. RDE-treated extracts showed less than 50% virus inhibition in *in vitro* micro-inhibition assay at a concentration of 25 µg/mL whereas native extracts which were not treated with RDE showed significant antiviral activity at the same concentration. Deactivation of sialic acid mimics that were originally present in the extract may be the reason for this significant drop in virus inhibition, though anti-influenza activity was observed at other concentrations tested (data not shown). Therefore, a potential synergistic effect of components apart from those that are HAI active may be present in the plant extracts. Further studies to investigate if there is any synergistic effect of neuraminidase inhibitory activity and hemagglutination inhibition exhibited by the plant extracts will be performed.

Apart from inhibiting hemagglutinin and neuraminidase, the plant extracts could also have affected other proteins in the virus including nucleoprotein, RNA polymerase, matrix protein1, nuclear export protein and non-structural protein 1 that play major roles during virus replication. Individual extract components could attack several targets, operating in a supportive agonistic, synergistic approach, called “synergistic multi target effects” [40]. The antiviral efficacy not being affected by temperature or trypsin treatment suggests that the compound(s) of interest may not be proteinaceous. It is worthwhile noting that minor variations were evident with the activity of extracts for different batches; this could possibly result from seasonal changes in the composition of plant extracts collected at different times or through the collection and extraction processes. For instance, extract 8 did not demonstrate HI activity in the most recent batch that was used to study the effects of trypsin treatment upon the antiviral activity of extracts, but the preliminary chemical fraction of extract 8 demonstrated HI activity in one of the fractions. This process is still under study. Also, the concentration at which HI activity was shown to be present for extracts 41/42 and 43 were different for the most recent batch unlike the results discussed in Figure 6. HI activity was present between 50–100 µg/mL for the most recent batch of 41/42 and 43 with HI activity being lost at concentrations less than 50 µg/mL. This phenomenon needs to be further examined.

The anti-influenza effects of HI extracts have not been published previously, though some plants belonging to the same genus have been reported to show antimicrobial activity [47]–[49]. Bioassay-guided fractionation of HI extracts (8, 41, 42 and 43) coupled with HPLC and GC-MS techniques are currently being undertaken. *In vivo* animal model studies that support the activity of extracts against influenza virus should also be performed.

The results presented in this study suggest that plants with reported medicinal properties could be a potential source for new antiviral drugs. The plant extracts investigated could serve as promising candidates for the development of third generation anti-influenza drugs, thereby challenging the neuraminidase drug resistant viruses in an attempt to safeguard human health and the global economy.
